# The breeding zone in a colonial marine invertebrate influences larval sensitivity to low oxygen at a micro-spatial scale

**DOI:** 10.1098/rspb.2025.0448

**Published:** 2025-07-02

**Authors:** Marcelo E. Lagos, Natalia Albarrán-Mélzer, Juan Diego Gaitán-Espitia

**Affiliations:** ^1^ The Swire Institute of Marine Science and School of Biological Sciences, The University of Hong Kong, Hong Kong; ^2^ Institute for Climate and Carbon Neutrality, The University of Hong Kong, Hong Kong

**Keywords:** *Bugula neritina*, ocean deoxygenation, oxygen, larvae settlement, hypoxia, marine invertebrates

## Abstract

In marine benthic environments, oxygen availability is highly variable across temporal and spatial scales. Such variability generates heterogeneous microhabitats in which organisms experience marked changes from saturated (i.e. normoxic) to anoxic conditions. For sessile colonial species, fine spatial differences in oxygen availability can trigger intra-colony phenotypic differences, influencing the overall colony performance/fitness. Here, we assessed the extent to which intra-colony differences in oxygen regimens influence biological characteristics in adult and larval stages of the colonial bryozoan *Bugula neritina*. For this, we measured the critical sensitivity to low oxygen for upper and lower zones of adult colonies and their larvae. We also measured larval swimming–exploring behaviour and settlement under hypoxia and normoxia. Although the results show similar intra-colony tolerances in the adults, differences were found in their larvae. While the lower zones of the colonies showed higher tolerant larvae, the upper zones had larvae with higher sensitivity and a tendency to avoid low-oxygen microhabitats. These larvae settle more quickly and in greater numbers compared with their lower-zone counterparts. Our results suggest that intra-colony differences in sensitivity to low-oxygen conditions (particularly during larval stages) can be important regulators of ecological processes (e.g. recruitment) and the resilience of benthic colonial species in deoxygenated oceans.

## 1. Introduction

Oxygen availability is crucial for the correct functioning, performance and fitness of marine animals. Exposure to suboptimal oxygen levels (e.g. hypoxia) can trigger negative effects on their physiology, development, settlement patterns, survival and the overall ecological dynamics of marine communities [1–4]. These suboptimal conditions are regularly experienced in benthic systems, in which oxygen levels are highly heterogeneous across temporal and spatial scales due to the interaction of biotic (e.g. community respiration) and abiotic (e.g. temperature) factors [5–7]. Such heterogeneity is also influenced by the oxygen demands of benthic animals (particularly dense aggregation of sessile invertebrates), the microbial activity and the complex topography of benthic systems. In consequence, oxygen levels in these habitats can change from normoxic to anoxic conditions even within a few centimetres, exposing marine animals to natural, but contrasting oxygen conditions at short spatial scales [5–7]. This natural spatial–temporal heterogeneity in oxygen availability can be intensified by anthropogenic pressures (e.g. eutrophication), particularly in coastal areas in which the construction of artificial structures (e.g. marinas, piers and pontoons) reduces water flow, and stimulates the generation of hypoxic and anoxic microhabitats [7,8]. However, it remains unclear to what extent these microhabitat differences in oxygen availability influence the physiology and ecological dynamics of benthic marine animals. In fact, the understanding of such effects has been mostly derived from studies at large spatial scales (e.g. dead zones; eutrophic areas) [9,10], while only few studies have emphasized the relevance of hypoxia at the microhabitat scale [5–7]. Such changes are expected to be driven by the increasing urbanization of coastal areas and the continued effects of climate change, aspects that have profound implications for coastal biodiversity [7,11]. Therefore, it is imperative to address this major knowledge gap, considering the projected intensification of hypoxia events in benthic systems [4,9].

Organismal responses to low-oxygen stress are diverse and vary among animals depending on their physiological tolerances and their different lifestyles [12,13]. Mobile species can seek more suitable areas and escape from stressful low-oxygen conditions [14,15]. In contrast, due to their limited mobility, sessile species require other types of behavioural and physiological adjustments to respond and persist in their habitats [16,17]. Such adjustments and the whole-organism tolerance to low oxygen are influenced by the size, morphology, aerobic scope and life history of benthic sessile animals [7,18,19]. For invertebrate colonial species, these characteristics and the overall responses to low-oxygen stress differ based on their geometric configuration [7]. Flat (two-dimensional) sessile colonies living in direct contact with the oxygen-depleted boundary layer exhibit greater resistance to low oxygen levels compared with erected (three-dimensional) species [5]. In the latter, gradients of oxygen availability are experienced along the colony in which zooids from the upper region have access to more oxygenated waters compared with their counterparts in the lower region of the colonies [5,7,20]. Given that colonial species are clonal, it would be expected that zooids from the same colony would display comparable responses and vulnerability to environmental stress (e.g. hypoxia) [21]. However, physiological acclimatization during colony formation can potentially induce intra-clonal differences in tolerances and the responses to stress across various locations within a colony [22–24]. These differences would be reflected in higher tolerances to low oxygen for the segments of adult colonies that are closer to or inside of the oxygen-depleted boundary layer (e.g. lower zone), compared with those segments that are more frequently exposed to water rich in oxygen (e.g. upper zone). In this way, phenotypic plasticity during larvae development can also play an important role in modulating similar intra-colony differences, particularly in invertebrate marine larvae, considering their higher sensitivity to stress [1,25], and the important contribution of the maternal/parental environment for their development [26].

Here, we hypothesize that larvae originating from the lower segments of a colony may exhibit lower sensitivity to hypoxia compared with their counterparts from the upper segments. These intra-colonial differences in larval sensitivities to low-oxygen conditions could influence larval behaviour (e.g. swimming, settlement) and their settlement selection [27–29]. Using this theoretical framework, we assessed the extent to which normoxic and hypoxic conditions influence intra-colony physiological differences and behavioural responses in adult colonies and larvae of the marine bryozoan *Bugula neritina* (Linnaeus, 1758; WoRMS: https://www.marinespecies.org/).

## 2. Material and methods

### (a) Colonies collection and general methods

*Bugula neritina* (hereafter *Bugula*) is a worldwide distributed, cryptic arborescent bryozoan that inhabits submerged hard substrates at shallow depths, typically associated with artificial structures (marinas, pontoons and jetties) [30,31]. *Bugula* releases ‘lecithotrophic’ larvae that are fully competent to settle immediately [30,31]. Thanks to this characteristic, *Bugula* is considered a good model for physiological and larval behavioural studies [32–35]. In Hong Kong, this species displays seasonal phenology with presence between November and March (winter months) in waters characterized by dissolved oxygen (DO) levels of 6.9 ± 0.9 (mg l^−1^; mean ± s.d.) and 91.2 ± 10 (% saturation; mean ± s.d.) (electronic supplementary materials) (Hong Kong Environmental Protection Department: https://cd.epic.epd.gov.hk/EPICRIVER/marine). For this study, mature colonies of *Bugula*, of around 9 cm length, were collected from floating piers located in Lamma Island, Hong Kong SAR, China (22°12′31.47″N, 114°07′56.49″E), between December 2022 and January 2023. Colonies were transported to the School of Biological Sciences, at The University of Hong Kong, where they were maintained in total darkness for 2 days to later induce spawning of larvae. Colonies were kept in aquaria with constant aerated seawater (autoclaved, 0.2 mm filtered) under controlled conditions (temperature: 22°C; salinity: 31‰). Then, colonies were randomly assigned to two different experimental groups for the physiology and the behavioural/settlement experiments. In both groups, colonies were cut and divided into three horizontal equidistant segments (3 cm). Only the upper and lower segments of the colonies were used for the experiments under laboratory-controlled conditions as described above. For larval rearing, the upper and lower segments (coming from 15 mature colonies) were separately placed inside transparent plastic boxes (2 l) filled with fully oxygen-saturated seawater. Then, colony segments were exposed to bright light to induce spawning before each experimental run [31,35,36]. Larvae released (typically within 10 min) were randomly collected from the pool of larvae by pipetting and assigned to each experimental chamber. Additionally, 20 larvae from each treatment were randomly measured (length and width; stereo microscope at 10×), to calculate their approximated volume and integrate it into equation (2.1); see §2b.

### (b) Estimation of sensitivity to low-oxygen conditions (CCO_2_)

The oxygen consumption (VO_2_) was measured using two different closed respirometry systems (for colony segments and larvae), with non-consumptive O_2_. Colony segments were introduced into 2 ml glass vials with hermetic caps (*n* = 24 per treatment). The vials had a magnetic bar inside to keep constant water recirculation using magnetic stirrers (Wiggens S-1, Beijing, China). The vials were connected to a four-channel fibre optic oxygen meter Oxy-4 SMA (G3) (PreSens GmbH, Regensburg, Germany). The 48 colony segments were tested in runs of four or five samples per day (5 days in total for each colony section), with each run lasting approximately 3–4 h. During the assessment, two blank vials were randomly interspersed to provide background respiration rates of the test water (i.e. containing water but no colony segments) [7,20].

For larvae oxygen consumption, pooled groups of 10 larvae were introduced into 80 μl chambers of a glass microplate, hermetically sealed with adhesive clear polyester films for Loligo System (*n* = 11 per treatment). Then, VO_2_ was assessed using a 24-channel PreSens Sensor Dish Reader SDR2 (Loligo Systems Aps, Tjele, Denmark), with four blanks (i.e. no larvae) [37,38]. This procedure was repeated during three experimental runs (four samples each day), lasting approximately 8–10 h each run. For both respirometer systems, the calibration was done with air-saturated seawater (100% AS) and saturated water with NaSO_3_ (0%AS).

The rates of oxygen consumption (VO_2_) were calculated with the following equation [5]:


(2.1) $$VO_{2}=-[(m_{a}-m_{c})/100]\;\times \;V\;\times \;\beta O_{2}.$$

With *m_a_* representing the slope for the relationship between the oxygen level (% air saturation) with time (h), for the vials containing organisms; *m_c_* is the slope for the relationship between oxygen level (% air saturation) and time (h), for the control vial; *V* is the volume of the chamber minus the volume of the organisms; and *β*O_2_ (5.1 ml l^−1^ at 22.0°C) is the oxygen capacitance of air-saturated seawater [39]. In the case of the larval VO_2_, values were divided by 10 (which is the total amount of larvae per vial) to get individual oxygen consumption [33]. After the measurements, the colony segments were placed in an oven for 2 days at 60°C to obtain dry biomass.

A common way to estimate the sensitivity to low oxygen is by measuring the ‘critical oxygen level’ through the *P*_crit_ (a parameter that can be used to represent the critical partial pressure [40–42] or the critical concentration of oxygen [6]). This point represents the level of O_2_ below which the metabolic rate of an animal becomes oxy-conformer [43]. Below that point, metabolic rate decreases, anaerobic pathways become more important and oxygen conditions are considered physiologically stressful [40,42,44]. The lower the critical value, the lower the sensitivity to low oxygen. However, some species present curvilinear relationships between the oxygen consumption rate and the oxygen availability (as in our case; see §3). This means that there is no clear point where the organisms transition from a perfect oxy-regulator to an oxy-conformer. To assess this trait, the oxygen consumption data were transformed to the relative $\dot{V}$O_2_ so all individuals present a value bounded between 0 and 1 (0−1) [7,45]. Then, we estimated the CCO_2_ (a proxy for *P*_crit_, equivalent to *K*_*m*_ [46]), by fitting the data to a Michaelis–Menten function [7,46] as follows:


(2.2) $$\dot{V}O_{2}=(O_{2max}\times CO_{2})/(CCO_{2}+CO_{2})$$

where $\dot{V}$O_2max_ is an asymptotic $\dot{V}$O_2_, CO_2_ is the oxygen level in the chamber, CCO_2_ is the value of oxygen level, where $\dot{V}$O_*2*_ = $\dot{V}$O_2max_/2. For detailed information about the assumptions of this model check Lagos *et al.* [7].

### (c) Behaviour and settlement experiments

All experimental surfaces used in the pre-settlement and settlement behaviour trials were sanded and immersed in non-filtered, aerated seawater for one week (31‰ salinity and 22°C) to develop biofilms [32]. During each experimental run (aquaria of 5 l, filled with filtered seawater), the oxygen levels were manipulated by bubbling nitrogen gas, and monitored using fibre-optic sensors connected to an oxygen meter Oxy-4 SMA (G3) (PreSens GmbH, Regensburg, Germany) [31,36], and then transferred to the experimental chambers. Our previous exploratory test did not show any variation in the pH (8.1) after injecting nitrogen into the water. The factorial experimental design included oxygen levels (high oxygen: 100% air saturation; low oxygen: 10% air saturation) and larvae breeding zone (upper and lower zone) as the two main factors. The oxygen levels were selected according to previous publications [31,36], with the low level representing some of the extreme oxygen conditions that these animals experience in the field [5,7,47]. Each experiment was conducted in two experimental runs (days), with 10 replications each run.

*Swimming and exploring behaviour. Bugula* larvae show behaviours characterized as ‘swimming’ and ‘exploring’. Swimming behaviour is identified as erratic and quick movement through the water column. Exploring behaviour, on the other hand, represents a fine-scale searching pattern identified by the adoption of a stationary position or slow movement on the bottom that occurs prior to settlement. Usually, larvae start the cycle through a general exploration of the habitat by swimming. When the habitat seems optimal, larvae go to the bottom and explore. If the larvae do not settle, they will go up to swim again. This cycle is repeated until the larvae decide where to finally settle [34,48]. In this experiment, we tested the effect of oxygen content on larval behaviour coming from the upper and lower zones of the colonies. Here, a single randomly selected larva was transferred into a petri dish (3.5 cm diameter, 1.1 cm depth) filled with seawater at the corresponding experimental oxygen level. Then, the behaviour was examined under a microscope for 5 min, recording the time spent by the larva exploring and swimming (*n* = 20 for each treatment).

*Settlement time*. In this experiment, single randomly selected larvae were individually transferred into 1.5 ml centrifuge tubes (*n* = 20 for each of the four treatments), filled with seawater at both experimental oxygen levels. Then, we measured the time until the larvae settled within a total period of 5 min.

*Total settlement*. For each experimental treatment, five larvae were transferred to a petri dish (3.5 cm diameter, 1.1 cm depth), with 20 replications for each treatment. The total number of larvae settled was measured in 0 min old larvae (i.e. individuals pipetted into the experimental surface immediately after spawning from the colony). Finally, the experiment was repeated using 60 min old larvae [31,33,36].

### (d) Statistical analysis

All data are presented as mean ± s.e. The assumptions of homoscedasticity and residual normality were tested using the Shapiro–Wilk, complemented by residual fitted plots for homoscedasticity and *Q*–*Q* plots for normality. Swimming proportion and total settlement were arcsine transformed to improve model fit and the distribution of residuals. Such approach is a standard procedure when analysing proportional data in ecology [49]. The effects of colony zone and day (fixed factors) on CCO_2_ of larvae and colony segments were tested using a two-way ANOVA. For the behavioural experiments, a three-way ANOVA was used to test the effects of the fixed factors (i.e. colony zone, oxygen content and day) and their interactions on swimming behaviour, settlement time and total settlement, followed by a Tukey’s honest significant difference test [50]. All analyses were conducted with R (v. 2022.12), using the ‘drc’ package for the Michaelis–Menten models [51]. All scripts are provided as electronic supplementary material.

## 3. Results

### (a) Estimation of sensitivity to low-oxygen conditions (CCO_2_)

**Figure 1.**
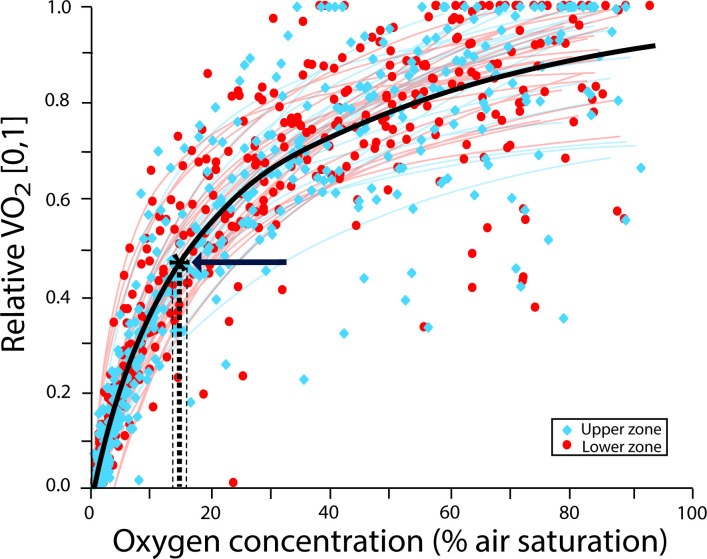
Plots of oxygen level (% air saturation) with relative oxygen consumption rate ($\dot{V}$O_2_, 0–1) of upper (blue squares) and lower zones (red circles) of mature colonies of the bryozoan *B. neritina*. Each dataset is fitted to a Michaelis–Menten function, where the red lines correspond to the upper segments of colonies and the blue lines to the lower segments of colonies. An overall model including all sets of data is represented (black line). The intersection of dashed lines (highlighted with an arrow) with the *x*-axis shows the CCO_2_ value (average ± s.e.).

**Figure 2.**
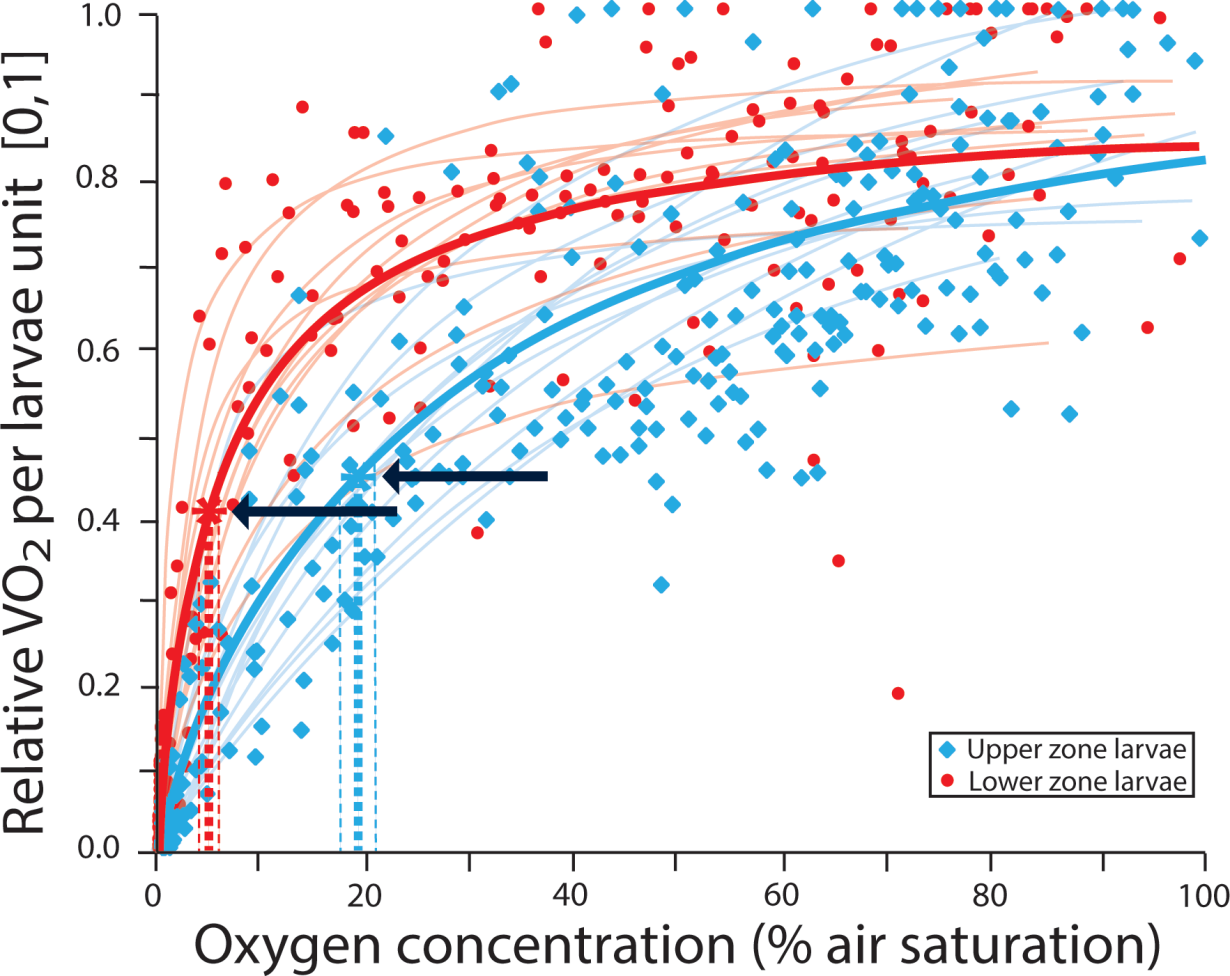
Plots of oxygen level (% air saturation) with relative oxygen consumption rate ($\dot{V}$O_2_, 0–1) of larvae breed in upper (blue squares ) and lower (red circles) zones, within colonies of the bryozoan *B. neritina*. Each dataset is fitted to a Michaelis–Menten function, where the blue lines correspond to larvae from upper zones and the red lines to larvae bred in the lower zone of the colonies. For larvae from each zone, an overall model including all sets of data are represented (red and blue bold lines). The intersection of dashed lines (highlighted with an arrow) with the *x*-axis shows the CCO_2_ value (average ± s.e.).

The upper and lower zones of the colonies showed no significant difference in the CCO_2_ (*F*_(1,46*)*_ = 0.6218, *p* = 0.43), with an average of 14.57 ± 0.69% air saturation (figure 1). However, a significant effect was found for the larvae depending on their breeding zone within the colony (*F*_(1,20)_= 23.299, *p* ≤ 0.001). Larvae from the lower zone showed significantly lower CCO_2_ (6.38 ± 2.95% air saturation) compared with their upper-zone counterparts (19.13 ± 5.85% air saturation) (figure 2). In both cases, there was no significant effect of the experimental run (random factor) with a *X*^2^ = 0.99 for the colony segments and *X*^2^ = 0.14 for larvae. There was a significant difference in the dried mass of the colonies segments used in the measurements (*F*_(1,46)_ = 5.1898, *p* = 0.02), with an average dry mass of 0.033 ± 0.001 g for the upper segments of the colony and 0.039 ± 0.011 g for the lower segments. Moreover, there was significant difference in the volume of the larvae (*F*_(1,38)_ = 47.564, *p* < 0.001) between the upper (mean volume: 0.08 ± 0.003 mm^3^) and the lower (mean volume: 0.05 ± 0.002 mm^3^) zones of the colony.

### (b) Behaviour and settlement experiments

#### (i) Swimming and exploring behaviour

**Figure 3.**
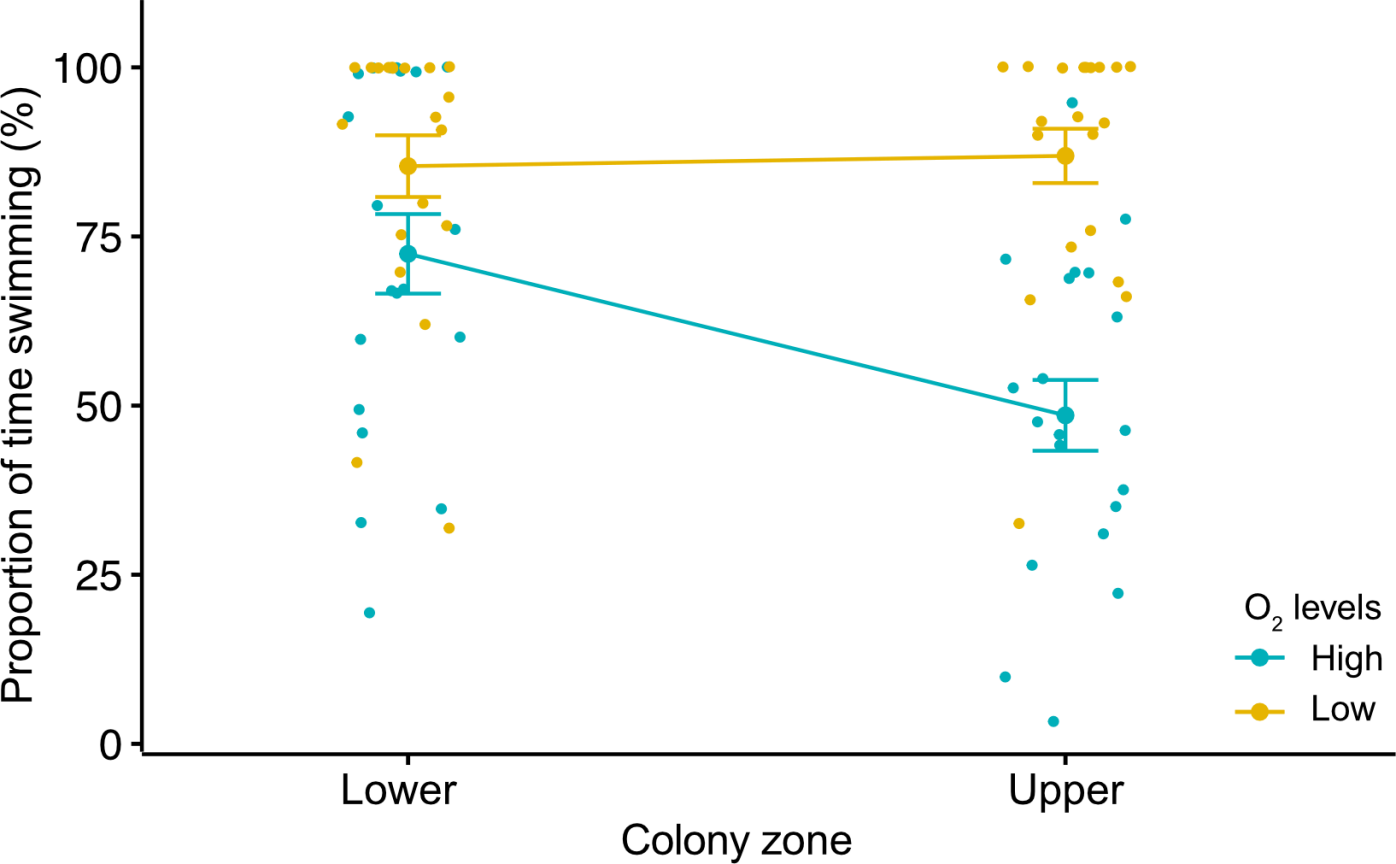
Main and interactive effects of colony zone and oxygen levels on the proportion of time swimming (%) of the bryozoan *B. neritina*. The jitter plot shows: (i) significant differences within the O_2_ level treatment for larvae bred in the upper zones; (ii) significant differences within the colony zone treatment for larvae under the high and low O_2_ level; and (iii) a significant interactive effect (differences between high O_2_ larvae from the upper zone with the low O_2_ larvae from the lower zone of the colony).

Main and interactive effects of the oxygen levels and the breeding zone on larval behaviour were detected (table 1A), with no influence of the experimental run (random factor; *X*^2^ = 0.99). Larvae bred in the upper zone of the colonies under high oxygen conditions showed the lowest proportion of time swimming (mean: 50.74 ± 5.23%). Larvae from this treatment showed significant differences compared with those from the lower zone under high- and low--oxygen conditions (Tukey < 0.05, in both cases). The latter group had the higher proportion of time swimming (approx. 85% of the time; figure 3).

**Table 1.** ANOVA for the effects of colony zone, oxygen level and their interaction on: (A) swimming time, (B) settlement time, (C) total settlement of 0 min old larvae and (D) total settlement of 60 min old larvae, in the bryozoan *B. neritina*.

	source	sum sq	d.f.	mean sq	*F*	*p*
A	‘swimming time’					
	colony zone	0.515	1	0.515	5.181	*0.025*
	oxygen level	2.600	1	2.600	26.161	*<0.001*
	colony zone: oxygen level	0.616	1	0.616	6.196	*0.014*
	residuals	7.555	76	0.099		
B	‘settlement time’					
	colony zone	8242	1	8242	0.681	0.411
	oxygen level	335 664	1	335 664	27.747	*<0.001*
	colony zone: oxygen level	57 138	1	57 138	4.723	*0.032*
	residuals	919 393	76	12 097		
C	‘total settlement (0 min)’					
	colony zone	0.040	1	0.040	0.340	0.561
	oxygen level	3.652	1	3.652	30.784	*<0.001*
	zone: oxygen level	1.722	1	1.722	14.515	*<0.001*
	residuals	9.017	76	0.118		
D	‘total settlement (60 min)’					
	colony zone	0.520	1	0.520	7.068	*0.009*
	oxygen level	0.211	1	0.211	2.870	0.094
	colony zone: oxygen level	0.500	1	0.500	6.794	*0.011*
	residuals	5.5913	76	0.073		

#### (ii) Settlement time

**Figure 4.**
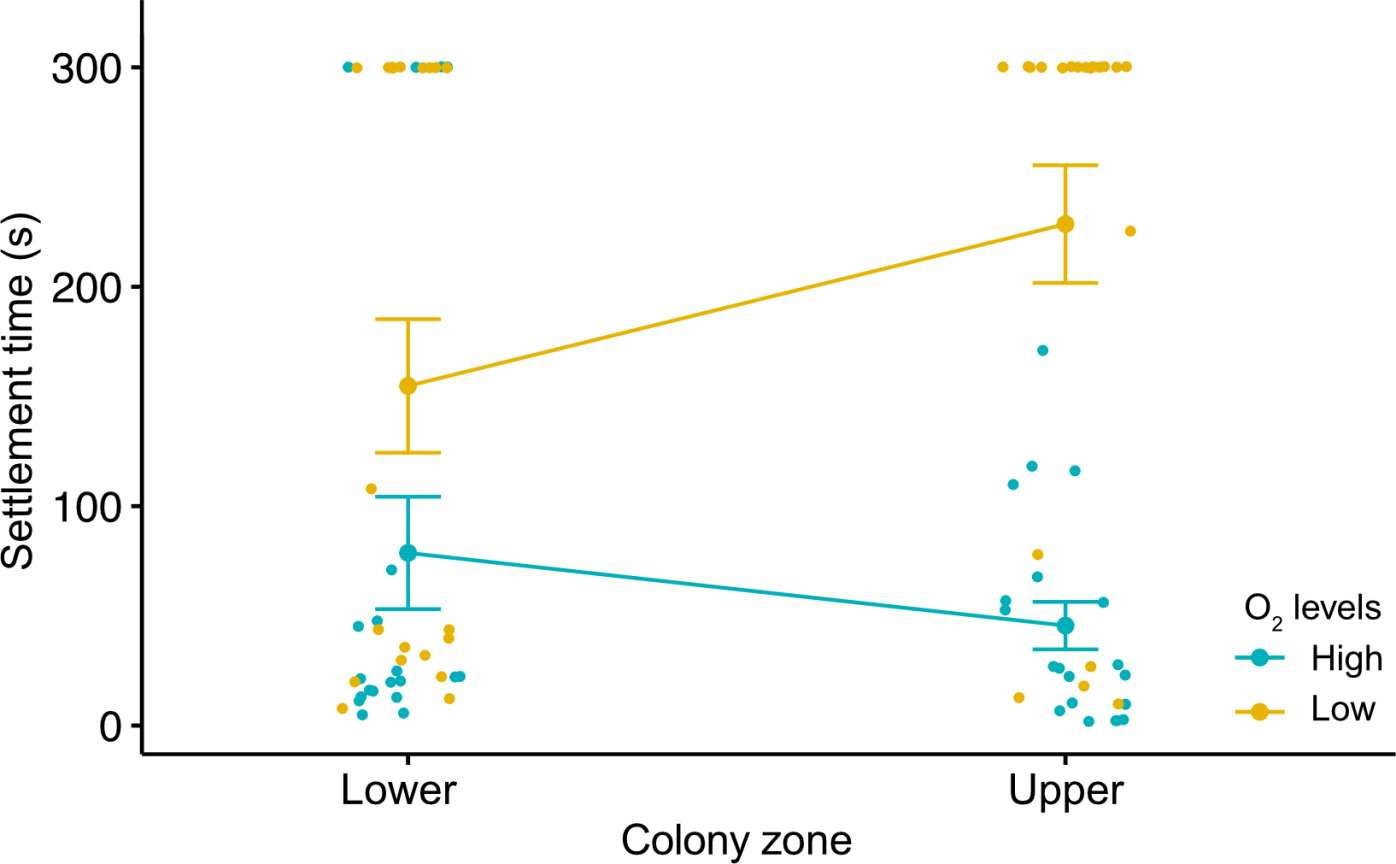
Main and interactive effects of colony zone and oxygen levels on the larval settlement time (s) of the bryozoan *B. neritina*. The jitter plot shows: (i) no main effect of the colony zone treatment; (ii) significant main effect of the O_2_ level treatment for larvae bred in the upper zones; and (iii) significant interactive effects across levels of the two factors.

There was a significant main effect of the oxygen level, but no effect was detected for the breeding zone (table 1B, figure 4). These factors showed a significant interaction with no effect of the experimental run (random factor: *X^2^* = 0.05). Irrespective of the breeding zone, larvae exhibited the quickest settlement (approx. 45–78 s; figure 4) under high oxygen conditions. While the lower-zone larvae showed no significant differences in settlement time between oxygen levels (Tukey = 0.135), there were significant differences for the upper-zone larvae (Tukey < 0.005; figure 4), with the slowest response (mean: 119 ± 26.82 s) under hypoxia conditions.

#### (iii) Total settlement

**Figure 5.**
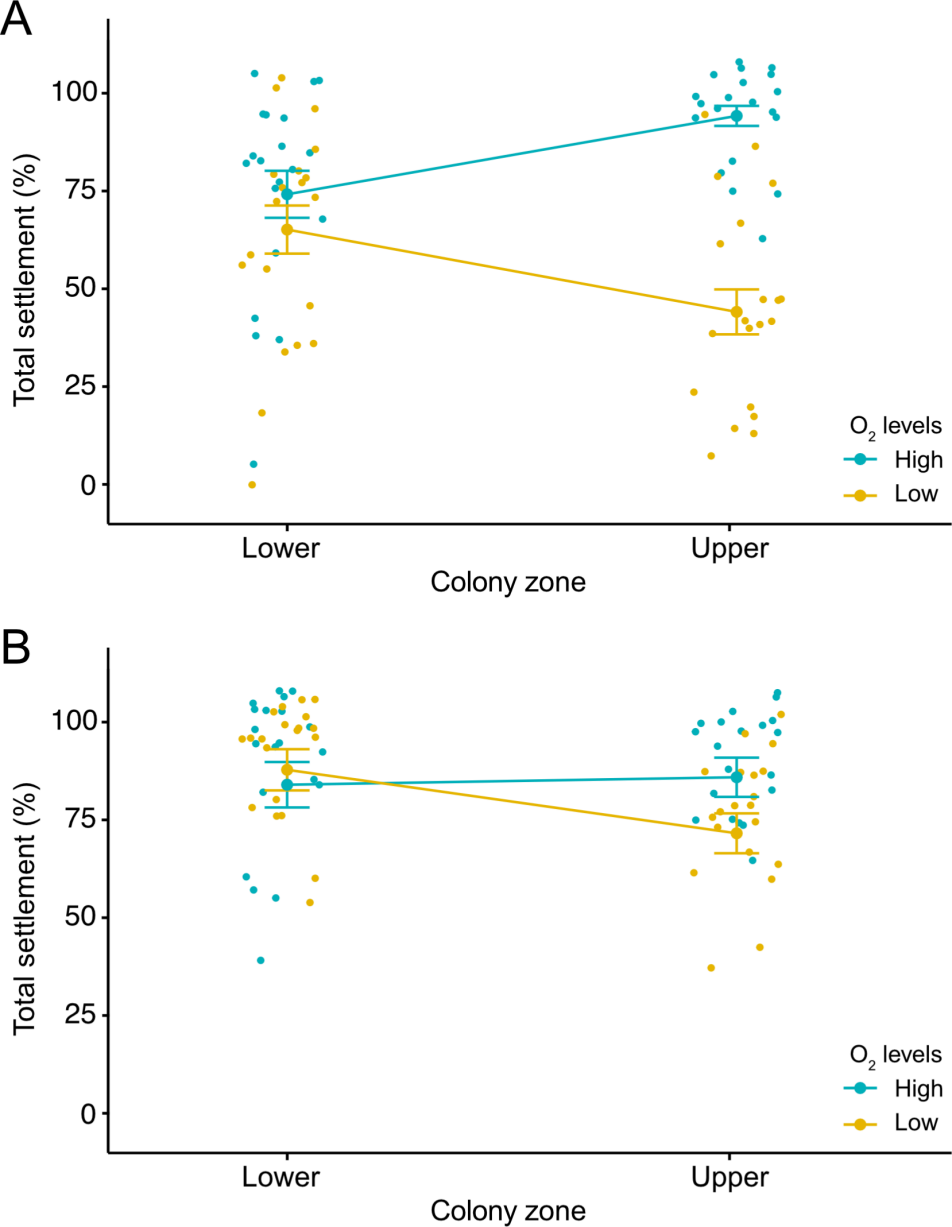
Jitter plots for the settled larvae (%) bred in lower and upper zones of mature colonies of the bryozoan *B. neritina*. (A) For the 0 min old larvae, the plot shows: (i) no main effect of the colony zone treatment; (ii) significant main effect of the O_2_ level treatment for larvae bred in the upper zones; and (iii) significant interactive effects across levels of the two factors. (B) For the 60 min old larvae, the plot shows: (i) no main effect of the O_2_ level treatment; and (ii) significant main effect of the colony zone and its interaction with the O_2_ level effects across levels of the two factors.

There was no significant effect of the breeding zone on total settlement for the 0 min old larvae. However, there was a significant effect of the oxygen level and the interaction of this factor with the breeding zone (table 1C, figure 5A). While no significant differences in settlement were found for the lower-zone larvae between oxygen levels (Tukey = 0.61, figure 5A), there were significant differences for their upper-zone counterparts (Tukey < 0.001). The highest (94.0 ± 2.55%) and lowest (44.0 ± 5.73%) settlements were documented in this group under high- and low-oxygen conditions, respectively (figure 5A). For the 60 min old larvae, significant effects of the breeding zone and the interaction of this factor with oxygen level were detected (table 1D). For this trait, the upper-zone larvae exposed to low oxygen levels showed the lowest total settlement (75.0 ± 3.80%), with significant differences when compared with all the other treatment combinations (Tukey < 0.05, figure 5B). Similar to the 0 min old larvae, no significant differences were found between oxygen conditions for the lower-zone larvae (Tukey = 0.91, figure 5B). No significant effect of the experimental run (random factor) was found for the 0 min old (*X^2^* = 0.15) and the 60 min old (*X^2^* = 0.95) larvae.

## 4. Discussion

Spatial and temporal variation in oxygen availability in marine benthic systems can generate heterogeneous oxygen seascapes that influence the distribution, vulnerability and physiology of sessile colonial species. For these organisms, settlement selection is one of the ecological processes affected by low-oxygen microhabitats in which the ability of the larvae to read environmental cues is significantly reduced [31,36,47]. It remains unclear, however, whether and how differential exposure to oxygen conditions among colonial organisms influence such responses, inducing intra-colony differences in physiological sensitivity to hypoxia. In this study, we assessed this research gap, exploring the overall influence of the larval breeding zones in colonial marine species, using the bryozoan *B. neritina* as a model system. Studying the effects of hypoxia in different zones of the colonies (upper and lower zones) sheds light on the importance of understanding stress at small spatial scales (i.e. microhabitat).

Physiological differences in stress responses within different parts of a colony have been observed in colonial organisms such as corals [22]. Despite having genetic homogeneity among polyps, each part of the colony exhibits different patterns of functional regulation that vary according to the differential exposure to stress [22]. Contrary to these expectations, upper and lower zones within adult colonies of *B. neritina* showed similar sensitivities to hypoxia (CCO_2_), despite their minor differences in dry mass. Such lack of influence of body size/mass on CCO_2_ has been previously documented across a range of invertebrate species [5,7,52], for which the metabolic rates and gas exchange capacities scale similarly with size/mass [53]. In our study, the mean CCO_2_ value found for *B. neritina* colonies (14.57 ± 0.69% air saturation) was similar to other *Bugula* species and *B. neritina* populations from Australia [5,7], and suggests high resistance to low-oxygen conditions [5,7]. In general, colonial bryozoans like *Bugula* are considered tolerant to hypoxia and can persist in hypoxic environments [5,7,52]. Therefore, the similar response of both zones of the colony may be due to the general tolerance to low oxygen of the species and its relatively low metabolic demands [20]. As not all species exhibit this type of responses and tolerances, it is important to conduct more species-specific studies to understand the role of ecological and evolutionary backgrounds shaping variability in the physiological responses to hypoxia within colonial organisms. Variability in the sensitivity (and vulnerability) to low oxygen may arise from energetic differences through development, particularly in early ontogenetic stages [54]. To our knowledge, such variability has only been previously documented in one study for marine invertebrate larvae [54]. Using different larval stages of the *Haliotis rubra* and *Haliotis laevigata*, authors found significant intra- and inter-species variation in low-oxygen sensitivity (*P*_crit_: 14.1−18.6% air saturation) [54]. For colonial organisms, such variation is hypothesized to operate at micro-spatial scales (i.e. intra-colony) driven by microhabitat differences in oxygen availability [7], potentially influencing larval behaviour (e.g. swimming, settlement) and their settlement selection [27–29]. Our study supports this hypothesis showing that phenotypic variation in low-oxygen sensitivity can be observed at the intra-individual level for colonial erected (three-dimensional) species, in which larvae bred in the lower zones of the colonies are less sensitive to low-oxygen conditions (i.e. lowest CCO_2_; 6.38 ± 2.95% air saturation) compared with their upper-zone counterparts (CCO_2_; 18.68 ± 2.38% air saturation). These intra-colony differences in larval sensitivity to hypoxia were reflected in the pre-settlement and settlement behaviour of the larvae, with variations in their performance within the spawning zone. In general, larvae from the upper zone performed better under normoxic conditions but displayed greater sensitivity to hypoxia (swim more, have a slower settlement and exhibit a lower total settlement) than larvae from the lower zone.

The traditional way of assessing settlement success (i.e. by only measuring final settlement and recruitment in response to a particular environmental stressor) has important limitations. For this trait, it is also fundamental to consider the selection behaviour of the larvae under stress, as their ability to select a suitable habitat is constrained by exogenic and endogenic factors [32,55,56]. Using this approach, our results indicate that larvae from the lower segments of the colonies are less efficient, regardless of the oxygen conditions. Their swimming time (70–90% of the total time) was equivalent to the time for the upper-zone larvae under hypoxic conditions. Larvae from upper zones of the colonies were more selective and had shorter swimming time (approx. 50%) in high oxygen conditions. A similar response was found for the settlement time, showing that larvae from the lower zones of the colonies were less sensitive to oxygen levels compared with those from the upper zones, which settle within seconds. The contrasting responses of upper-zone larvae to oxygen conditions suggest that, in nature, they have a higher ability to detect low oxygen and keep swimming, increasing their chances of finding and settling in a high-quality habitat rich in oxygen [5–7]. On the other hand, larvae bred in the lower segments of the colony probably settle in any place regardless of the oxygen conditions, as they are less oxygen selective.

Several studies have shown that acclimatization of organisms to specific environmental conditions can affect morphological, physiological and behavioural responses to environmental stressors [57–60]. This suggests that the upper- and lower-zone larvae of *Bugula* colonies may have different acclimatization responses to oxygen availability, resulting in different sensitivities. It is possible that the lower-zone larvae are more tolerant of hypoxia due to their exposure to lower oxygen levels during their embryonic development compared with the upper-zone larvae (i.e. developmental plasticity). In this case, the microhabitat background of the colonial segment may underpin the phenotypic differences detected among larvae generated from genetically identical zooids [61]. Furthermore, the maternal/parental effects may have played a critical role in shaping the intra-colonial phenotypic differences shown in the larvae [62]. These effects are known to influence various aspects of larval development, including gene expression, epigenetic modifications, nutrient allocation and the physiological responses of offspring to environmental stressors, including hypoxia [63–65]. Further research is needed to determine the underlying mechanisms that contribute to the observed intra-colony differences in their vulnerability to hypoxia, and the extent of the carryover effects (i.e. within/trans-generational) induced by the microhabitat and the maternal/parental environment.

In our study, the total settlement of recently hatched larvae was significantly influenced by the combined effects of oxygen level and the breeding zone of the larvae. Larvae from the upper zone had high selectivity for high oxygen conditions (i.e. settlement 94.00 ± 2.55%), and showed reduced settlement capacity under hypoxia (i.e. 44.00 ± 5.73%). This reduced capacity seems to be associated with some degree of loss in the interpretation efficiency of environmental cues, as they have a similar proportion of settlement in all treatments. Similarly, *Bugula* larvae showed a reduced efficiency of habitat selection as they aged, evidencing an increasingly ‘desperate’ settlement behaviour [55,66,67]. Swimming more, delaying settlement and larvae getting ‘desperate’ to settle are all behavioural responses with deep implications for *Bugula* larvae, considering their lecithotrophic nature and their limited energy budget. Therefore, each second they spend swimming under low-oxygen conditions, represents less energy available for survival and growth in the future [32,68]. For larvae, high selectivity is beneficial as this behaviour enhances the probability of choosing settlement habitats with good quality, avoiding strong competitors and maximizing their fitness [27,36,69]. However, there are trade-offs associated with high selectivity as swimming is an energetically expensive activity considering the negative buoyancy of *Bugula* larvae [33,70]. Individuals that spend more time swimming (e.g. 60% of the time) have elevated energetic costs (around 0.4 J h^-1^) compared with larvae with shorter swimming times (approx. 20% time invested, 0.05 J h^-1^) [33]. Therefore, the avoidance of hypoxia and the delay of settlement represent a trade-off in terms of the reduction of energy reserves and the energy allocation for post-metamorphic growth, leading to the formation of smaller and less successful colonies [34,56,71,72]. These effects can be more accentuated in larvae from the upper zones of the colonies, as they avoid hypoxia in higher proportion than larvae from the lower zones.

Benthic marine biodiversity is under high pressure due to the increase in the frequency and magnitude of hypoxic events and the scale of low-oxygen zones. Therefore, it is fundamental to understand the capacity of these organisms to adjust their responses (e.g. behaviour, physiology) to cope with such environmental threats. Although we know that this capacity can vary among species and populations, our study has provided novel insights demonstrating that it can also vary within individuals (for colonial species), at least in part, as a consequence of oxygen differences at the microhabitat scale. Overall, our findings contribute to a better understanding of the fundamental role of hypoxia at small spatial scales (i.e. microhabitat) shaping ecological dynamics, phenotypic differences and the regulation of functional responses in colonial organisms. This has important implications for predicting the impacts of anthropogenic activities, for the conservation and management of benthic systems. This information should be considered in future studies of larval ecology, since the breeding zone of the colonies can significantly influence larval physiological sensitivity, tolerance, performance and behaviour in hypoxic systems. Future studies should also assess the extent to which intrinsic differences in biological characteristics within colonial organisms (e.g. age of the zooids in the different colony sections; size and number of zooids) contribute to the observed intra-individual phenotypic differences in hypoxia sensitivity and vulnerability.

## Data Availability

All data and scripts are available from HKU Figshare Data repository [73].

## References

[1] Alter K, Paschke K, Gebauer P, Cumillaf JP, Pörtner HO. 2015 Differential physiological responses to oxygen availability in early life stages of decapods developing in distinct environments. Mar. Biol. **162**, 1111–1124. (10.1007/s00227-015-2654-4)

[2] Auel H, Verheye HM. 2007 Hypoxia tolerance in the copepod Calanoides carinatus and the effect of an intermediate oxygen minimum layer on copepod vertical distribution in the northern Benguela Current upwelling system and the Angola–Benguela Front. J. Exp. Mar. Biol. Ecol. **352**, 234–243. (10.1016/j.jembe.2007.07.020)

[3] Lagos M, Cáceres CW, Lardies MA. 2014 Geographic variation in acid–base balance of the intertidal crustacean Cyclograpsus cinereus (Decapoda, Grapsidae) during air exposure. J. Mar. Biol. Assoc. UK **94**, 159–165. (10.1017/s0025315413001264)

[4] Diaz RJ. 2001 Overview of hypoxia around the world. J. Environ. Qual. **30**, 275–281. (10.2134/jeq2001.302275x) 11285887

[5] Ferguson N, White CR, Marshall DJ. 2013 Competition in benthic marine invertebrates: the unrecognized role of exploitative competition for oxygen. Ecology **94**, 126–135. (10.1890/12-0795.1) 23600247

[6] Candy AS *et al*. 2023 Small-scale oxygen distribution patterns in a coral reef. Front. Mar. Sci. **10**, 1135686. (10.3389/fmars.2023.1135686)

[7] Lagos ME, Barneche DR, White CR, Marshall DJ. 2017 Do low oxygen environments facilitate marine invasions? Relative tolerance of native and invasive species to low oxygen conditions. Glob. Chang. Biol. **23**, 2321–2330. (10.1111/gcb.13668) 28212460

[8] Lin QY, Yu S. 2018 Losses of natural coastal wetlands by land conversion and ecological degradation in the urbanizing Chinese coast. Sci. Rep. **8**, 15046. (10.1038/s41598-018-33406-x) 30301927 PMC6177474

[9] Diaz RJ, Rosenberg R. 2008 Spreading dead zones and consequences for marine ecosystems. Science **321**, 926–929. (10.2307/20144596) 18703733

[10] Altieri A, Harrison S, Seemann J, Collin R, Diaz RJ, Knowlton N. 2017 Tropical dead zones and mass mortalities on coral reefs. Proc. Natl Acad. Sci. USA **114**, 201621517. (10.1073/pnas.1621517114) PMC5389270 28320966

[11] Roman MR *et al*. 2024 Reviews and syntheses: biological indicators of low-oxygen stress in marine water-breathing animals. Biogeosciences **21**, 4975–5004. (10.5194/bg-21-4975-2024)

[12] Abele D, Kruppe M, Philipp EER, Brey T. 2010 Mantle cavity water oxygen partial (Po_2_) pressure in marine molluscs aligns with lifestyle. Can. J. Fish. Aquat. Sci. **67**, 977–986. (10.1139/f10-035)

[13] Melzner F, Gutowska MA, Langenbuch M, Dupont S, Lucassen M, Thorndyke MC, Bleich M, Pörtner HO. 2009 Physiological basis for high CO_2_ tolerance in marine ectothermic animals: pre-adaptation through lifestyle and ontogeny? Biogeosciences **6**, 2313–2331. (10.5194/bg-6-2313-2009)

[14] Richardson J, Williams EK, Hickey CW. 2001 Avoidance behaviour of freshwater fish and shrimp exposed to ammonia and low dissolved oxygen separately and in combination. N. Z. J. Mar. Freshw. Res. **35**, 625–633. (10.1080/00288330.2001.9517028)

[15] Yu HL, Fang GJ, Rose KA, Lin JZ, Feng J, Wang HY, Cao QX, Tang YL, Zhang T. 2023 Effects of habitat usage on hypoxia avoidance behavior and exposure in reef-dependent marine coastal species. Front. Mar. Sci. **10**. (10.3389/fmars.2023.1109523)

[16] Pan J, Cheng FP, Yu F. 2018 The diel vertical migration of zooplankton in the hypoxia area observed by video plankton recorder. Indian J. Geo Mar. Sci. **47**, 1353–1363.

[17] Broszeit S, Davenport J, Bredendieck K, Harman L, McAllen R. 2013 Seasonal oxygen-driven migration of mobile benthic fauna affected by natural water column stratification. Estuar. Coast. Shelf Sci. **125**, 36–42. (10.1016/j.ecss.2013.03.020)

[18] Frank L, Serafy J, Grosell M. 2023 A large aerobic scope and complex regulatory abilities confer hypoxia tolerance in larval toadfish, Opsanus beta, across a wide thermal range. Sci. Total Environ. **899**, 165491. (10.1016/j.scitotenv.2023.165491) 37453709

[19] Hughes DJ, Alderdice R, Cooney C, Kühl M, Pernice M, Voolstra CR, Suggett DJ. 2020 Coral reef survival under accelerating ocean deoxygenation. Nat. Clim. Chang. **10**, 296–307. (10.1038/s41558-020-0737-9)

[20] Lagos ME, White CR, Marshall DJ. 2017 Do invasive species live faster? Mass‐specific metabolic rate depends on growth form and invasion status. Funct. Ecol. **31**, 2080–2086. (10.1111/1365-2435.12913)

[21] Lasker HR, Coffroth MA. 1999 Responses of clonal reef taxa to environmental change. Am. Zool. **39**, 92–103.

[22] Drake JL, Malik A, Popovits Y, Yosef O, Shemesh E, Stolarski J, Tchernov D, Sher D, Mass T. 2021 Physiological and transcriptomic variability indicative of differences in key functions within a single coral colony. Front. Mar. Sci. **8**, 685876. (10.3389/fmars.2021.685876)

[23] Chang S, Elkins C, Alley M, Eaton J, Monismitha S. 2009 Flow inside a coral colony measured using magnetic resonance velocimetry. Limnol. Oceanogr. **54**, 1819–1827. (10.4319/lo.2009.54.5.1819)

[24] Hughes DJ. 1992 Genotype-environment interactions and relative clonal fitness in a marine bryozoan. J. Anim. Ecol. **61**, 291–306. (10.2307/5322)

[25] Pardieck RA, Orth RJ, Diaz RJ, Lipcius RN. 1999 Ontogenetic changes in habitat use by postlarvae and young juveniles of the blue crab. Mar. Ecol. Prog. Ser. **186**, 227–238. (10.3354/meps186227)

[26] Proulx SR, Teotónio H. 2017 What kind of maternal effects can be selected for in fluctuating environments? Am. Nat. **189**, E118–E137. (10.1086/691423) 28514627

[27] Grosberg RK. 1981 Competitive ability influences habitat choice in marine invertebrates. Nature **290**, 700–702. (10.1038/290700a0)

[28] Mundy CN, Babcock RC. 1998 Role of light intensity and spectral quality in coral settlement: implications for depth-dependent settlement? J. Exp. Mar. Biol. Ecol. **223**, 235–255. (10.1016/s0022-0981(97)00167-6)

[29] Thiyagarajan V. 2010 A review on the role of chemical cues in habitat selection by barnacles: new insights from larval proteomics. J. Exp. Mar. Biol. Ecol. **392**, 22–36. (10.1016/j.jembe.2010.04.030)

[30] Jaeckle W. 1994 Rates of energy consumption and acquisition by lecithotrophic larvae of Bugula neritina (Bryozoa: Cheilostomata). Scholarship **119**, 517–523. (10.1007/BF00354313)

[31] Lagos ME, White CR, Marshall DJ. 2015 Avoiding low-oxygen environments: oxytaxis as a mechanism of habitat selection in a marine invertebrate. Mar. Ecol. Prog. Ser. **540**, 99–107. (10.3354/meps11509)

[32] Marshall DJ, Keough MJ. 2003 Variation in the dispersal potential of non-feeding invertebrate larvae: the desperate larva hypothesis and larval size. Mar. Ecol. Prog. Ser. **255**, 145–153. (10.3354/meps255145)

[33] Pinochet J, Urbina MA, Lagos ME. 2020 Marine invertebrate larvae love plastics: habitat selection and settlement on artificial substrates. Environ. Pollut. **257**, 113571. (10.1016/j.envpol.2019.113571) 31733954

[34] Burgess SC, Simon PH, Marshall DJ. 2009 Pre-settlement behavior in larval bryozoans: the roles of larval age and size. Biol. Bull. **216**, 344–354. (10.2307/25548165) 19556599

[35] Strathmann MF. 1987 Reproduction and development of marine invertebrates of the northern Pacific Coast: data and methods for the study of eggs, embryos, and larvae. Seattle, WA: University of Washington Press.

[36] Lagos ME, White CR, Marshall DJ. 2016 Biofilm history and oxygen availability interact to affect habitat selection in a marine invertebrate. Biofouling **32**, 645–655. (10.1080/08927014.2016.1178725) 27169475

[37] Pettersen AK, White CR, Marshall DJ. 2015 Why does offspring size affect performance? Integrating metabolic scaling with life-history theory. Proc. R. Soc. B **282**, 20151946. (10.1098/rspb.2015.1946) PMC4685814 26559952

[38] Pettersen AK, White CR, Marshall DJ. 2016 Metabolic rate covaries with fitness and the pace of the life history in the field. Proc. R. Soc. B **283**, 20160323. (10.1098/rspb.2016.0323) PMC4892794 27226476

[39] Cameron JN. 1986 Principles of physiological measurement. London, UK: Academic Press. (10.1016/B978-0-12-156955-6.50007-4)

[40] Rogers NJ, Urbina MA, Reardon EE, McKenzie DJ, Wilsonl RW. 2016 A new analysis of hypoxia tolerance in fishes using a database of critical oxygen level (P_crit_). Conserv. Physiol. **4**, cow012. (10.1093/conphys/cow012) 27293760 PMC4849809

[41] Marshall DJ, Bode M, White CR. 2013 Estimating physiological tolerances – a comparison of traditional approaches to nonlinear regression techniques. J. Exp. Biol. **216**, 2176–2182. (10.1242/jeb.085712) 23470657

[42] Hochachka PW, Somero GN. 2002 Biochemical adaptation: mechanism and process in physiological evolution. New York, NY: Oxford University Press.

[43] Portner HO, Grieshaber MK. 1993 Critical Po2(s) in oxyconforming and oxyregulating animals: gas-exchange, metabolic-rate and the mode of energy-production. In The vertebrate gas transport cascade adaptations to environment and mode of life (ed. J Bicudo), pp. 330–357. Boca Raton, FL: CRC Press.

[44] Armstrong W, Webb T, Darwent M, Beckett PM. 2009 Measuring and interpreting respiratory critical oxygen pressures in roots. Ann. Bot. **103**, 281–293. (10.1093/aob/mcn177) 18819952 PMC2707311

[45] Pearson K. 2014 The grammar of science. Cambridge, UK: Cambridge University Press.

[46] Wood CM. 2018 The fallacy of the P_crit_ – are there more useful alternatives? J. Exp. Biol **221**, jeb163717. (10.1242/jeb.163717) 30420494

[47] Jorissen H, Nugues MM. 2021 Coral larvae avoid substratum exploration and settlement in low-oxygen environments. Coral Reefs **40**, 31–39. (10.1007/s00338-020-02013-6)

[48] Walters LJ, Miron G, Bourget E. 1999 Endoscopic observations of invertebrate larval substratum exploration and settlement. Mar. Ecol. Prog. Ser. **182**, 95–108. (10.3354/meps182095)

[49] Warton DI, Hui FK. 2011 The arcsine is asinine: the analysis of proportions in ecology. Ecology **92**, 3–10. (10.1890/10-0340.1) 21560670

[50] Quinn GP, Keough MJ. 2002 Experimental design and data analysis for biologists. New York, NY: Cambridge University Press.

[51] Ritz C, Baty F, Streibig JC, Gerhard D. 2016 Dose-response analysis using R. PLoS One **10**, e0146021. (10.1371/journal.pone.0146021) PMC4696819 26717316

[52] Byers JE, Blaze JA, Dodd AC, Hall HL, Gribben PE. 2023 Exotic asphyxiation: interactions between invasive species and hypoxia. Biol. Rev. **98**, 150–167. (10.1111/brv.12900) 36097368 PMC10087183

[53] Lease HM, Klok CJ, Kaiser A, Harrison JF. 2012 Body size is not critical for critical PO_2_ in scarabaeid and tenebrionid beetles. J. Exp. Biol. **215**, 2524–2533. (10.1242/jeb.057141) 22723492

[54] Alter K, Andrewartha SJ, Elliott NG. 2016 Hatchery conditions do not negatively impact respiratory response of early life-stage development in Australian hybrid abalone. J. Shellfish Res. **35**, 585–591. (10.2983/035.035.0303)

[55] Toonen RJ, Pawlik JR. 2001 Settlement of the gregarious tube worm Hydroides dianthus (Polychaeta: Serpulidae). II. Testing the desperate larva hypothesis. Mar. Ecol. Prog. Ser. **224**, 115–131. (10.3354/meps224115)

[56] Cancino JM, Gallardo JA. 2004 Effects of delaying settlement on the life expectancy of the bryozoan Bugula flabellata (Bryozoa: Gymnolaemata). Rev. Chil. Hist. Nat. **77**, 227–234. (10.4067/S0716-078X2004000200002)

[57] Somero GN. 2010 The physiology of climate change: how potentials for acclimatization and genetic adaptation will determine ‘winners’ and ‘losers’. J. Exp. Biol. **213**, 912–920. (10.1242/jeb.037473) 20190116

[58] Tomanek L, Somero GN. 2002 Interspecific- and acclimation-induced variation in levels of heat-shock proteins 70 (hsp70) and 90 (hsp90) and heat-shock transcription factor-1 (HSF1) in congeneric marine snails (genus Tegula): implications for regulation of hsp gene expression. J. Exp. Biol. **205**, 677–685. (10.1242/jeb.205.5.677) 11907057

[59] Albarrán-Mélzer NC, Rangel Ruiz LJ, Benítez HA, Lagos ME. 2020 Can temperature shift morphological changes of invasive species? A morphometric approach on the shells of two tropical freshwater snail species. Hydrobiologia **847**, 151–160. (10.1007/s10750-019-04078-z)

[60] Marshall DJ, Brahim A, Mustapha N, Dong YW, Sinclair BJ. 2018 Substantial heat tolerance acclimation capacity in tropical thermophilic snails, but to what benefit? J. Exp. Biol. **221**, jeb187476. (10.1242/jeb.187476) 30291160

[61] Winston JE. 2010 Life in the colonies: learning the alien ways of colonial organisms. Integr. Comp. Biol. **50**, 919–933. (10.1093/icb/icq146) 21714171

[62] Zhang Y, Million WC, Ruggeri M, Kenkel CD. 2019 Family matters: variation in the physiology of brooded Porites astreoides larvae is driven by parent colony effects. Comp. Biochem. Physiol. A Mol. Integr. Physiol. **238**, 110562. (10.1016/j.cbpa.2019.110562) 31493555

[63] Badyaev AV, Uller T. 2009 Parental effects in ecology and evolution: mechanisms, processes and implications. Phil. Trans. R. Soc. B **364**, 1169–1177. (10.1098/rstb.2008.0302) 19324619 PMC2666689

[64] Mousseau TA, Fox CW. 1998 The adaptive significance of maternal effects. Trends Ecol. Evol. **13**, 403–407. (10.1016/s0169-5347(98)01472-4) 21238360

[65] Cameron H, Monro K, Marshall DJ. 2017 Should mothers provision their offspring equally? A manipulative field test. Ecol. Lett. **20**, 1025–1033. (10.1111/ele.12800) 28726317

[66] Knight-Jones EW. 1953 Decreased discrimination during setting after prolonged planktonic life in larvae of Spjrorbis borealis (Serpulidae). J. Mar. Biol. Assoc. UK **32**, 337–345. (10.1017/s0025315400014594)

[67] Elkin C, Marshall DJ. 2007 Desperate larvae: influence of deferred costs and habitat requirements on habitat selection. Mar. Ecol. Prog. Ser. **335**, 143–153. (10.3354/meps335143)

[68] Carrier TJ, Reitzel AM, Heyland A. 2017 Evolutionary ecology of marine invertebrate larvae. Oxford, UK: Oxford University Press.

[69] Jensen GC. 1989 Gregarious settlement by megalopae of the porcelain crabs Petrolisthes cinctipes (Randall) and P. eriomerus Stimpson. J. Exp. Mar. Biol. Ecol. **131**, 223–231. (10.1016/0022-0981(89)90114-7)

[70] Pires A, Woollacott RM. 1983 A direct and active influence of gravity on the behavior of a marine invertebrate larva. Science **220**, 731–733. (10.1126/science.220.4598.731) 17813877

[71] Wendt DE, Woollacott RM. 1999 Ontogenies of phototactic behavior and metamorphic competence in larvae of three species of Bugula (Bryozoa). Invertebr. Biol. **118**, 75–84. (10.2307/3226915)

[72] Marshall DJ, Pechenik JA, Keough MJ. 2003 Larval activity levels and delayed metamorphosis affect post-larval performance in the colonial ascidian Diplosoma listerianum. Mar. Ecol. Prog. Ser. **246**, 153–162. (10.3354/meps246153)

[73] Lagos M, Albarran-Melzer N, Gaitan-Espitia JD. 2025 Data for: the breeding zone in a colonial marine invertebrate influences larval sensitivity to low oxygen at a micro-spatial scale. HKU Data Repository. (10.25442/hku.24850704) PMC12213005 40592452

